# Polycaprolactone based pharmaceutical nanoemulsion loaded with acriflavine: optimization and *in vivo* burn wound healing activity

**DOI:** 10.1080/10717544.2022.2136783

**Published:** 2022-10-26

**Authors:** Touseef Nawaz, Muhammad Iqbal, Barkat Ali Khan, Naveed Ahmed, Asif Nawaz, Akhtar Rasul, Waleed Y. Rizg, Abdulmajeed M. Jali, Rayan A. Ahmed, Awaji Y. Safhi

**Affiliations:** a Drug Delivery and Cosmetic Lab (DDCL), Faculty of Pharmacy, Gomal University, Dera Ismail Khan, Pakistan; b Department of Pharmacy, Quaid-i-Azam University Islamabad, Pakistan; c Department of Pharmaceutics, Government College University, Faisalabad, Pakistan; d Department of Pharmaceutics, Faculty of Pharmacy, King Abdulaziz University, Jeddah, Saudi Arabia; e Center of Excellence for Drug Research and Pharmaceutical Industries, King Abdulaziz University, Jeddah, Saudi Arabia; f Department of Pharmacology and Toxicology, College of Pharmacy, Jazan, Saudi Arabia; g Department of Pharmaceutics, College of Pharmacy, Jazan University, Jazan, Saudi Arabia

**Keywords:** Nanoemulsion, burn wound, acriflavine, topical, drug delivery

## Abstract

Cutaneous burn wounds are a common and troublesome critical issue of public health. Over the last decade, many researchers have investigated the development of novel therapeutic modalities which are capable of fully regeneration and reinstatement of structure and function of the skin with no or limited scar formation. Novel pharmaceutical carriers are offering a potential platform to deliver the drug effectively and to overcome the limitation associated with conventional wound dressings. The aim of this study was to investigate a pharmaceutical acriflavine-loaded polycaprolactone nanoemulsion (ACR-PCL-NE) for burn wound healing. Nanoemulsion was prepared by using the double emulsion solvent evaporation technique and it was subjected to thermodynamic stability testing, droplet size, polydispersity, zeta potential, pH, and surface morphology analysis. The *in vivo* study was performed to evaluate the efficacy of nanoemulsion using Sprague-Dawley rats as an animal model. The results of this study revealed that the optimized nanoemulsion was stable and had desirable physicochemical properties. The pH was about 4.02 at 25 °C and the particle size was found to be in the range of 302 ± 4.62 nm while the zeta potential was −7.8 ± 1.22 mV and the polydispersity index of 0.221 ± 0.017. The wound regeneration process was evaluated *in vivo* by different techniques, the formulation group (FG) showed high wound healing potential as compared to the standard group (SD) and control group (CG). These findings reveal that this nanoemulsion formulation can be used effectively for wound healing.

## 1. Introduction

Burns wounds are more prevalent and critical care problems that need timely cures in order to prevent further complications. Over seven million patients are affected by acute and chronic full-thickness wounds in the US, which is a major source of mortality and morbidity. An estimated 500,000 people get their burn treatment each year in the USA, chronic and severely burn wound recovery is a slow and costly process because they need multiple treatments. The need for better and effective wound healing is increasing day by day (Murphy et al., 2017). Early and proper treatment means timely healing of wounds and high chances of survivability. A patient with early treatment recovers quickly with a fast wound cure and re-epithelialization (Lesher et al., 2011). There is a need for novel treatment procedures and effective products which have high clinical efficiency (Rahmanian-Schwarz et al., 2011).

Wound repair is a very complex and highly organized process consisting of the following phases; i.e., (i) hemostasis, (ii) inflammation, (iii) proliferation, and (iv) remodeling (maturation) (Singh et al., 2017a). In these phases, a series of dynamic and complex events occur, like vascularization, clothing, epithelialization, tissue granulation, wound contraction, and collagen synthesis (Alsarra, 2009; Choi et al., 2014). In the healing process, inflammatory cells of surrounding areas move toward the site of the wound, after this migration, the fibroblasts reappear and the process of collagen fibers formation gets started (Md et al., 2022). At the same time, new blood vessel formation starts, which helps in oxygen and nutrient supply to the affected area, and epithelial cells start to fill the affected area under the scab. In the final phase, new epithelial cells emerge and the wound-healing process gets completed (Castano et al., 2018).

Topical drug delivery systems are based on localized drug availability for therapeutic effects, and the drug is directly applied to the affected area for local or systemic effects. The delivery of the drug through skin, vigina, rectum, ophthalmic, and nasal routes may be used for the topical drug delivery system, though skin is easily accessible organ for topical preparations (Czajkowska-Kośnik et al., 2019; Bhowmik, 2012). Nanoemulsions are the dispersion of two immiscible liquids which are stabilized using different stabilizers. Nanoemulsions are characterized by; i) brawny stability ii) optically transparent appearance iii) high surface area per unit volume and iv) tuneful rheology (Yu et al., 2021b). High and low energy techniques may be used for nanoemulsions preparation, such as ultra-sonication, high-pressure homogenization, bubble burst method, and phase inversion temperature method (Singh et al., 2017b; Kumar et al., 2019). Usually, its particle size is less than 500 nm, which results in the hazy and clear nature of nanoemulsions. Nanoemulsions might have the same particle size as that of microemulsions, but they differ in thermodynamic stability and structural aspects (Zhao et al., 2020).

Acriflavine has many pharmacological properties, such as antibacterial, topical antiseptic, antiviral, and antifungal properties. It is also active against *Escherichia coli* (Hirota & Iijima, 1957; Das et al., 2017), drug-resistant *Staphylococcus aureus* (Mitsuhashi et al., 1963), and *Helicobacter pylori* (Tehlan et al., 2020). It can be used for burn wound healing (Asuquo et al., 2009). Polycaprolactone (PCL) is biodegradable polyester having good hydrophobicity with a glass transition temperature of −60 °C and it melts at 59–64 °C. PCL is available in molecular weights between 3000 and 90,000 g/mol and it can be divided into different grades on the basis of molecular weight. As the molecular weight increases the crystallinity of the polymer decreases. Low melting point, good solubility, and blend compatibility give this polymer a unique role in biomedical research (Mohamed & Yusoh, 2015). It is also used in the production of nanoparticles (Alex et al., 2016) and microparticles (Gurler et al., 2019) due to its long-term release rate, biocompatibility, and slow biodegradability.

The objectives of this work were to prepare and characterize acriflavine-loaded PCL nanoparticles (ACR-PCL-NE) for the management of burn wound healing and to evaluate the prepared formulation in vivo in an animal model. Acriflavine was used as a model drug, which was incorporated into polycaprolactone particles. Male Sprague-Dawley rats were used as an animal model to assess the efficacy of the prepared dosage form, for this purpose, wound contractions were measured after different intervals of time. ATR-FTIR study was performed to follow up the recovery rate of burn wounds using IR spectra. A tensile strength study was performed using a Universal Testing Machine (UTM).

## 2. Materials and methods

### 2.1. Materials

Acriflavine was obtained from Pharmawise Labs Pakistan. Polycaprolactone (PCL) MW= 1400 g/mol, Dichloromethane (DCM), and Polyvinyl alcohol (PVA) MW= 31,000 g/mol were obtained from Sigma Aldrich (Germany). Double distilled water was obtained from DDC lab Gomal University D.I.Khan Pakistan. High-speed homogenizer HG-150A Daihan scientific (South Korea) was used for homogenization. All chemicals used in this study were of analytical grade.

### 2.2. Preparation acriflavine loaded nanoemulsion

ACR-PCL-NE was prepared using a double emulsion solvent evaporation method as described by Miladi et al. (2015) with a little modification. The organic phase was formed by dissolving a 1.5 g of PCL in 4 ml DCM till clear solution was formed, and the aqueous phase of primary emulsion was formed by dissolving 20 mg acriflavine in double distilled water (0.5 ml), then both aqueous and organic phases were mixed together to form primary emulsion (W/O) and homogenized for 3 min with 3000 RPM. Then, the primary emulsion was added to secondary emulsion which was containing 0.5% PVA aqueous solution and homogenized for 5 min at 12,000 rpm to obtain a W/O/W double emulsion. The polymeric droplets solidify in the presence of an external aqueous phase to make a particulate system. Then the organic solvent was removed by using a rotary evaporator and the resultant particles were washed by centrifugation at 10,000 rpm for 10 min which were then freeze-dried and stored for further characterization.

### 2.3. *In vitro* characterization

#### 2.3.1. Stability study

The prepared nanoemulsion was subjected to thermodynamic stability studies according to the ICH guidelines (Muthu & Feng, 2009). The prepared formulation was stored at various temperatures, i.e., 8 °C, 25 °C, 40 °C and 40 °C + 75% relative humidity for a time period of 28 days. The pH, homogeneity, and phase separation of prepared formulations were noted periodically (Zahid et al., 2022).

#### 2.3.2. pH

pH is an important factor affecting the micro-components of the skin’s outer layer. The pH of the nanoemulsion must be compatible with the skin in order to avoid skin irritation. pH has a direct effect on physical barrier components molecular order, metabolic processes, and cutaneous microbiome (Wohlrab & Gebert, 2018). The pH of formulation was determined using a digital pH meter (PHS-3C, China). The pH of ACR-PCL-NE was checked at different time points (12 h, 24 h, 2 days, 3 days, 7 days, 14 days, 21 days, and 28 days) at 8 °C, 25 °C, 40 °C and 40 °C + 75% relative humidity (Khan et al., 2018).

#### 2.3.3. Phase separation studies

In order to select the optimized formulation, it must be stable when subjected to thermodynamic stability studies. To check the phase separation and turbidity in ACR-PCL-NE, it was evaluated at 8 °C, 25 °C, 40 °C and 40 °C + 75% relative humidity in triplicates. The selected optimized ACR-PCL-NE was then subjected to centrifugation at 5000 rpm for 30 min and checked for formulation stability and phase separation (Thomas et al., 2017; Batool et al., 2021).

#### 2.3.4. Particle size, zeta potential, and polydispersity

Particle size, zeta potential, and polydispersity of ACR-PCL-NE were analyzed using zetasizer (MADLS, Malvern, Grovewood Road, UK). Before the experimentation, isotonicity was adjusted by adding 1 mM NaCl to the ACR-PCL-NE. a dilution of 100 folds using deionized water was executed to dodge multiple scattering in the formulation. An angle of 90 was maintained for scattering at 25 ± 1 °C (Alshehri et al., 2020; Bibi et al., 2022). The particle size, zeta potential, and polydispersity of ACR-PCL-NE were monitored for 90 days (fresh, 30 days, 60 days, and 90 days). The ACR-PCL-NE was stored at 25 °C throughout the course (Romes et al., 2021; Asfour et al., 2022; Mushtaq et al., 2022).

#### 2.3.5. Surface morphology

Scanning electron microscope (SEM) was used to assess and visualize the morphology of ACR-PCL-NE. Priorly, ACR-PCL-NE was lyophilized using a freeze dryer (BK-FD 10 series, Biobase, China). After lyophilization of the formulation, ACR-PCL-NE was placed on aluminum stubs using adhesive double tape. ACR-PCL-NE was visualized using SEM (Carl-Zeiss Inc, Germany) to study the surface morphology of the formulation under high vacuum with a high accelerated voltage of 10 KV. The nanoemulsion particles were visualized at 500×, 2500×, 5000×, and 10,000× magnification (Din et al., 2017; Mahtab et al., 2020; Nawaz et al., 2021).

#### 2.3.6. *In vitro* drug release assay

ACR-PCL-NE of 1 ml volume was added to a dialysis bag with a length of 4 cm. The dialysis bag or tubing is made up of regenerated cellulose, which is treated physically and chemically to boost its resistance (MWCO 8 0 00 -∼ 14,000 Da) (SERVA, Heidelberg, Germany) having a pore size of 25 Å (Khaleeq et al., 2020). Both ends of dialysis tubing were clipped using dialysis tubing closures (Merck, Germany). The dialysis bag was pendented in 25 mL PBS at pH 7.4 and maintained at 37 ± 1 °C. After burn wound, the pH of the skin disrupts, exposing the more neutral pH, i.e., 7.4 of the underlying tissue, therefore, release study was performed at neutral pH (Jones et al., 2015). The dispersion was rotated at 200 rpm in a shaker (GFL Shaker, LABOTEC, Germany). A sample of 1 ml was withdrawn from the outer phase with a time interval of 0.5, 1, 1.5, 2, 4, 8,12, 16, 20, and 24 h and a fresh phosphate buffer of pH 7.4 was added back to the outer phase. Acriflavine concentration in a collected sample was determined using a spectrophotometer (Shimadzu, Japan) at 416 nm. A triplicate repetition was followed for all experiments (Din et al., 2019; Macedo et al., 2014; Krausz et al., 2015).

### 2.4. *In-vivo* burn wound studies

#### 2.4.1. Animal selection

Male Sprague-Dawley rats, 250 ± 10 g were obtained from the National Institute of Health (NIH) Pakistan and they were used. Rats were divided into 3 groups; control group (CG), standard group (SG) and formulation group (FG). They were housed and maintained at the animal house by the Faculty of Pharmacy at Gomal University in accordance with university guidelines for laboratory animal care and use, approved by university Ethical Review Board (ERB) Gomal University. All rats were given free access to water, food and fresh air throughout the experimental period and temperature was maintained at 26 °C with relative humidity of 60 ± 10% of the facility and 12 h alternate light and dark cycle. After completion of experimental readings, all rats were sacrificed by cervical dislocation method.

##### Ethical approval

The study was conducted according to the guidelines of the NIH, by the Institutional Ethical Review Board of Gomal University, Dera Ismail Khan, Pakistan under reference no. 117/ERB/GU dated 25 February 2021.

#### 2.4.2. Skin irritation test

Skin irritation test was performed according to the method described by Norisca et al. (Putriana & Husni, 2018). Prior to the test, 6 animals from each group were selected and caged in polypropylene cages with free access to water, fresh air, and standard laboratory diet. A single dose of standard formulation (Acriflavine®) and ACR-PCL-NE was applied to normal skin for 6 days to check for the development of any erythema.

### 2.5. Burn wound establishment

NIH Guide for care and use of laboratory animals (NIH publication No 18-23, 1985) USA and Guidelines of ethical review board (ERB) Gomal University (NO: 117/ERB/GU) were followed during animal care and experimentation. For burn wound formation in rats, the rats were anesthetized using ketamine and xylazine with a dose of 40 mg/kg and 5 mg/kg, respectively (Xu et al., 2007). After anesthesia, the back of the rat was shaved using a sharp razor and the skin was disinfected using 70% ethanol. A tube of 1 cm in diameter was placed on the shaved back of the rat and hot water (94 ± 1 °C) was poured into a tube placed on the rat abdomen for 15 seconds to form a second-degree burn wound. Then the formulation and standard preparations were applied for 14 days in all groups to access the wound size reduction. Additionally, ATR FTIR studies and tensile strength studies were performed to confirm the re-epithelialization process of wound (Alemdaroğlu et al., 2006; Alsarra, 2009).

### 2.6. Application of standard drug and formulated nanoemulsion (ACR-PCL-NE)

All 39 rats were divided into 3 groups: group 1 was the control group where no drug was applied, group 2 was the standard group where commercially available Acriflavine® (0.1%) was applied to the burn wound, and group 3 was the formulation group where ACR-PCL-NE were applied to burn wound. All dosage forms were applied once a day for 14 days. After application of dosage form, wounds were covered using non-adhering dressing and allowed to heal. Bandage replacement was done on a daily basis. The same procedure for replacing bandages was applied daily for the control group (Jamshaid et al., 2022).

### 2.7. Wound size contraction analysis

After application of the dosage form, the wound contraction of all animals of each group was measured regularly on the 3^rd^, 7^th^, 10^th^, and 14^th^ day of wound formation. Equation (1) was used to calculate % wound contraction in rat’s burn wound.

(1) $$\text{\% wound contraction}=\frac{A_{0}-A_{t}}{A_{0\;}}\;\times 100\;$$

where *A*_0_ is an area of the wound on day 1^st^, and *A*_t_ is an area of the wound on a specific day (Murphy et al., 2017).

**Figure 1.**
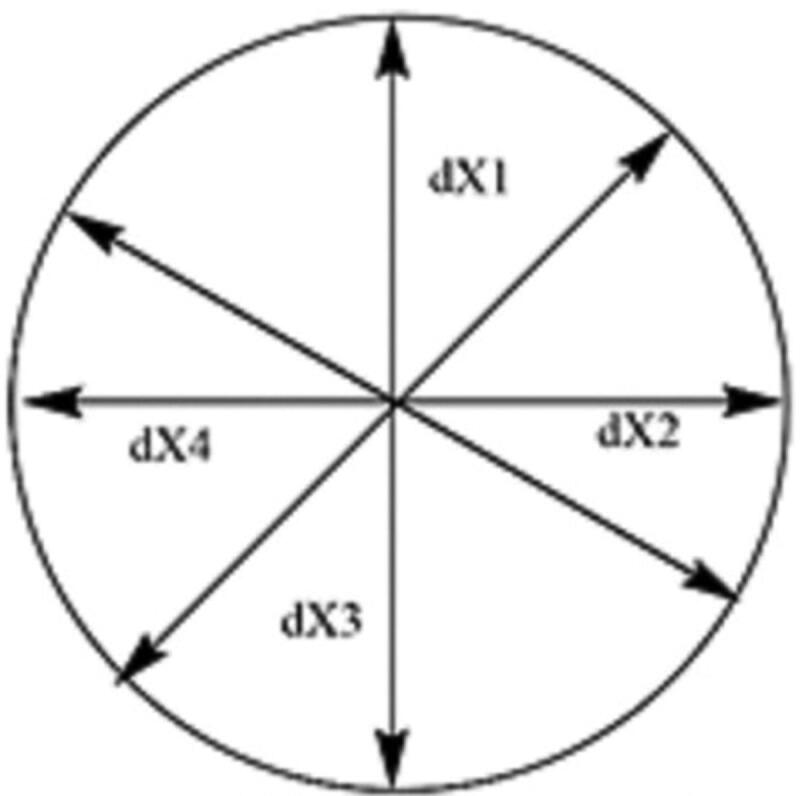
Calculation of burn wound area.

A caliper was used to measure the diameter of the produced burn wound. The procedure for measuring the diameter of the burn is shown in Figure 1, the area was circumscribed four times and the average of the four calculations was determined (Equation (2)).

Diameter of wound (dX) = $\frac{dX1+dX2+dX3+dX4}{4}$ (2).where dX was the diameter of the burn wound on a specific day.

Area was calculated from the diameter, *A* = π(*d*/2)^2^ (Silalahi & Surbakti, 2015).

### 2.8. ATR-FTIR burn wound analysis

The absorbance mode was used for the FTIR measurements. The Thermo Scientific Fourier transform spectrometer (iS50FT-IR, Waltham, Massachusetts, U.S.) has a KBr beam splitter and a DLaTGS (Deuterated Lanthanide Triglycine Sulfate) detector; the KBr window was used for the MAX IR microscope. The spectra were scanned at a resolution of 4 cm in the mid-IR band from 4000 to 400 cm^−1^. For each spectrum, 100 scans were coded, and the spectra were compared against the background spectrum (Ashtarinezhad et al., 2015; Rana et al., 2020).

#### 2.8.1. Data analysis and processing

FTIR is considered as an alternate method to analyze burn wound healing stages (Castro et al., 2018). The spectrophotometric peaks band changing in burn wound healing process was investigated. The burn wound areas were collected on 3^rd^, 7^th^, and 14th days after wound formation, for ATR-FTIR peaks analysis. The data were evaluated with the MainFTOS IR software’s procedures. After the full spectrum was baseline corrected, the spectra were standardized. The spectra were collected from burn wound portions of skin, and an average spectrum was calculated from all of the spectra readings of various animal groups (i.e., 30 for CG, 30 for SG and 30 for FG). Second-order derivatives were computed as well. The second derivative calculation improved spectral characteristics while simultaneously compensating for baseline changes. Various spectral characteristics were calculated and plotted against the x and y pixel coordinates using normalized absorbance spectra.

### 2.9. Skin tensile strength analysis

Universal testing machine (UTM) was used to measure skin and compressive strength. A graph was plotted between force and elongation. A formalin preserved rat skin of control, standard group, and formulation group was cut into 7 cm × 3 cm slices to be analyzed by UTM at Centralized Research Lab at the University of Peshawar. The thickness of the skin was 2.5 mm and a force (KN) of 1 KN till peak load reached to its maximum at speed of 1 mm/minute was applied (Brosh et al., 2004; Labroo et al., 2021).

### 2.10. Statistical analysis

All data were developed as the mean ± SD. Data analysis was transacted using two-way analysis of variance using GraphPad Prism 8.4® and Microsoft Excel 2016®.

## 3. Results and discussion

### 3.1. *In vitro* characterization

#### 3.1.1. pH

The normal pH of the skin falls between 3 and 6. The pH of optimized ACR-PCL-NE was determined using a digital pH meter (Vivoson, China). Table 1 shows the pH values of CR-PCL-NE at 8 °C, 25 °C, 40 °C and 40 °C + 75% relative humidity (RH) after 12 h, 24 h, 2 days, 3 days, 7 days, 14 days, 21 days, and 28 days. The pH of ACR-PCL-NE decreases slightly with time. Initially, the pH of fresh ACR-PCL-NE formulation was 4.54, which then changed to 3.98, 4.02, 3.70 and 3.81 at 8 °C, 25 °C, 40 °C and 40 °C ± 75% RH, respectively, on 28^th^ day. Statistically, the ACR-PCL-NE pH was insignificant with respect to time. This insignificance shows that the formulation was stable throughout the stability studies and also confirms that the ingredients of the formulation were also stable throughout the course. This concludes that there was no chemical ionization and degradation in ACR-PCL-NE during the test course.

**Table 1.** PH of ACR-PCL-NE at 8 °C, 25 °C, 40 °C and 40 °C ± 75% RH after 12 h, 24 h, 2 days, 3 days, 7 days, 14 days, 21 days, and 28 days.

	pH			
**Time intervals**	**8 °C**	**25 °C**	**40 °C**	**40 °C ± 75% RH**
0 h	4.54	4.54	4.54	4.54
12 h	4.54	4.52	4.49	4.52
24 h	4.51	4.52	4.46	4.47
2 days	4.36	4.39	4.41	4.43
3 days	4.14	4.30	4.33	4.36
7 days	4.10	4.20	4.16	4.21
14 days	4.06	4.16	4.01	4.09
21 days	4.02	4.10	3.76	3.91
28 days	3.98	4.02	3.70	3.81

#### 3.1.2. Phase separation studies

The ACR-PCL-NE stability and phase separation were checked at 8 °C, 25 °C, 40 °C and 40 °C ± 75% RH after incubation time the formulations were subjected to centrifugation at 5000 rpm for 10 minutes of centrifugation. No phase separation or change in color of the formulation was reported over time.

#### 3.1.3. Particle size, zeta potential, and polydispersity index

Nanoemulsion stability is an important factor which explains formulation shelf life. Inspecting mean particle size, zeta potential and PDI at 25 °C for 90 days is important for optimized nanoemulsion to find out the system thermodynamic stability against oswald ripening and coalescence. Table 2 shows the mean particle size, zeta potential and PDI of ACR-PCL-NE on day 0, day 30, day 60 and day 90. The stability of formulation is associated with ostwald ripening which leads to phase separation of the system. The increase in particle size to 422 nm ± 5.4 nm after 90 day was associated with both the phenomena; coalescence and ostwald ripening (Sulaiman et al., 2016).

**Table 2.** Mean particle size, zeta potential and PDI of ACR-PCL-NE on day 0, day 30, day 60 and day 90.

Days	Particle size (nm)	Zeta potential (mV)	PDI
0	302.2 ± 4.62	−7.8 ± 1.22	0.221 ± 0.017
30	345.5 ± 3.16	−5.72 ± 2.01	0.266 ± 0.013
60	391.7 ± 6.43	−4.63 ± 1.89	0.292 ± 0.018
90	422 ± 5.40	−3.74 ± 1.65	0.301 ± 0.021

The PDI of the system increased with the passage of time, on day 0, the ACR-PCL-NE, the PDI was 0.221 ± 0.017 and on day 90, it was 0.301 ± 0.021, this increase in PDI is associated with the coalescence of small particles to form large particles in the system. However, polyvinyl alcohol was used as stabilizer in order to stabilize the system and manimized the aggrigation and coalescence of particle. As a result the PDI (<0.35) was maintained in the acceptable range throughout 90 days (Pongsumpun et al., 2020; Romes et al., 2021).

The zeta potential of ACR-PCL-NE was found to be −7. 86 mV ± −1.22 mV on day 0 which decreases to −3.74 mV ± 1.65 mV on day 90 which is a very slight change. The zeta potential vales of the prepared formulation was very low which could be considered zero and it may be attributed to uncharged nature of PCL (Romes et al., 2021).

#### 3.1.4. Surface morphology

**Figure 2.**
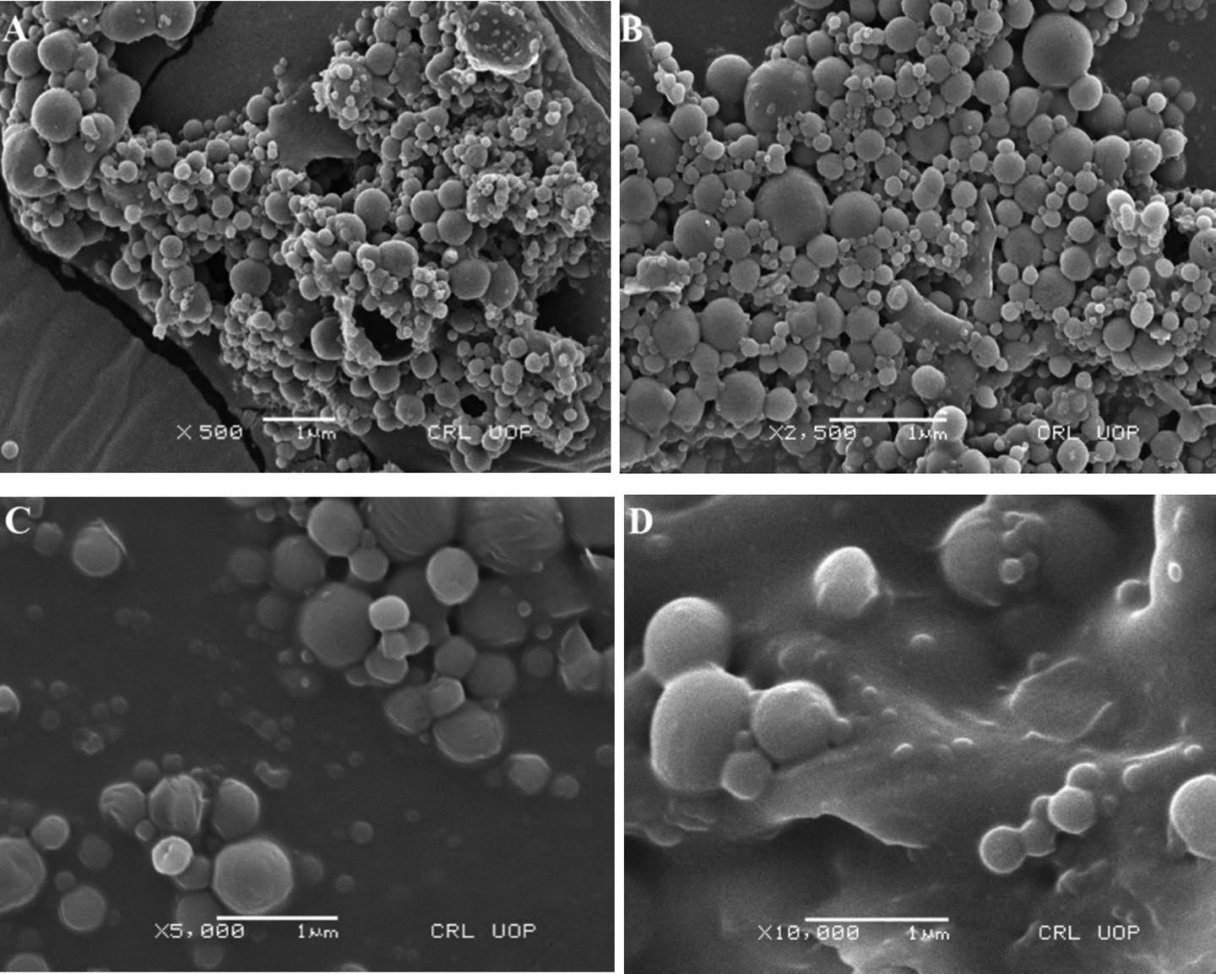
SEM micrographs of ACR-PC-NE at (A) 500×, (B) 2500×, (C) 5000×, and (D) 10,000× magnification.

SEM was used to study the surface morphology of ACR-PCL-NE prepared by using the double emulsion solvent evaporation method. SEM results confirmed the smooth, spherical structure of the polymeric nanoparticles as shown in Figure 2. SEM images also confirm interparticle bridging, which was due to PVA presence in the formulation, as it is challenging to washout PVA completely. The smooth surface of these particles also supports the assumption of acriflavine release by matrix erosion (Iqbal et al., 2015).

#### 3.1.5. *In vitro* release profile

**Figure 3.**
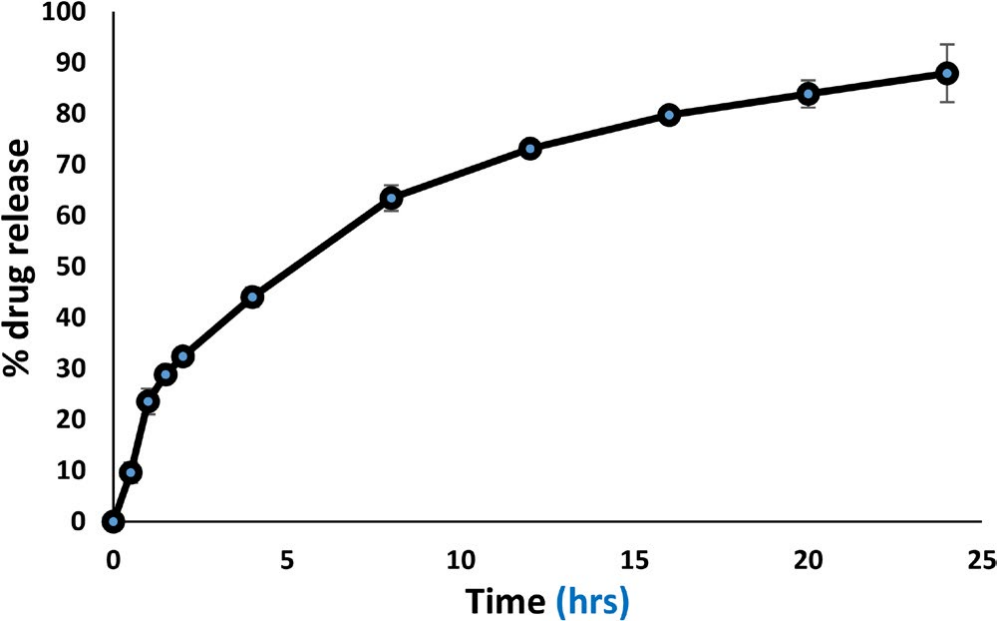
Cumulative % acriflavine release from ACR-PCL-NE.

An *in vitro* release study assay, acriflavine release from acriflavine solution and ACR-PCL-NE were evaluated using the dialysis bag method. Acriflavine solution in BPS pH 7.4 in dialysis bag showed a 97.8 ± 2.4% of release. Initially, the formulation exhibits fast and burst release followed by a controlled release for 24 h. About 87.91 ± 2.67% of acriflavine release was observed in 24 h (Figure 3). The initial burst release was due to some acriflavine presence on the surface of ACR-PCL-NE globules or dispersed in PVA which also have drug entrapment capabilities. The idea that the drug is ensnared within a nanoemulsion droplet cannot be ignored once there is a gradual release over time, since the cumulative release reached an almost steady state at the end of the experiment (Yu et al., 2021a; Kim et al., 2021).

### 3.2. *In vivo* characterization

#### 3.2.1. Skin irritation test

This test was repeated for 6 days and no erythema or redness was found as a result of the application of prepared formations. These results revealed that skin has no sensitivity or allergic response to the prepared formulations formulation

#### 3.2.2. % Wound contraction

**Figure 4.**
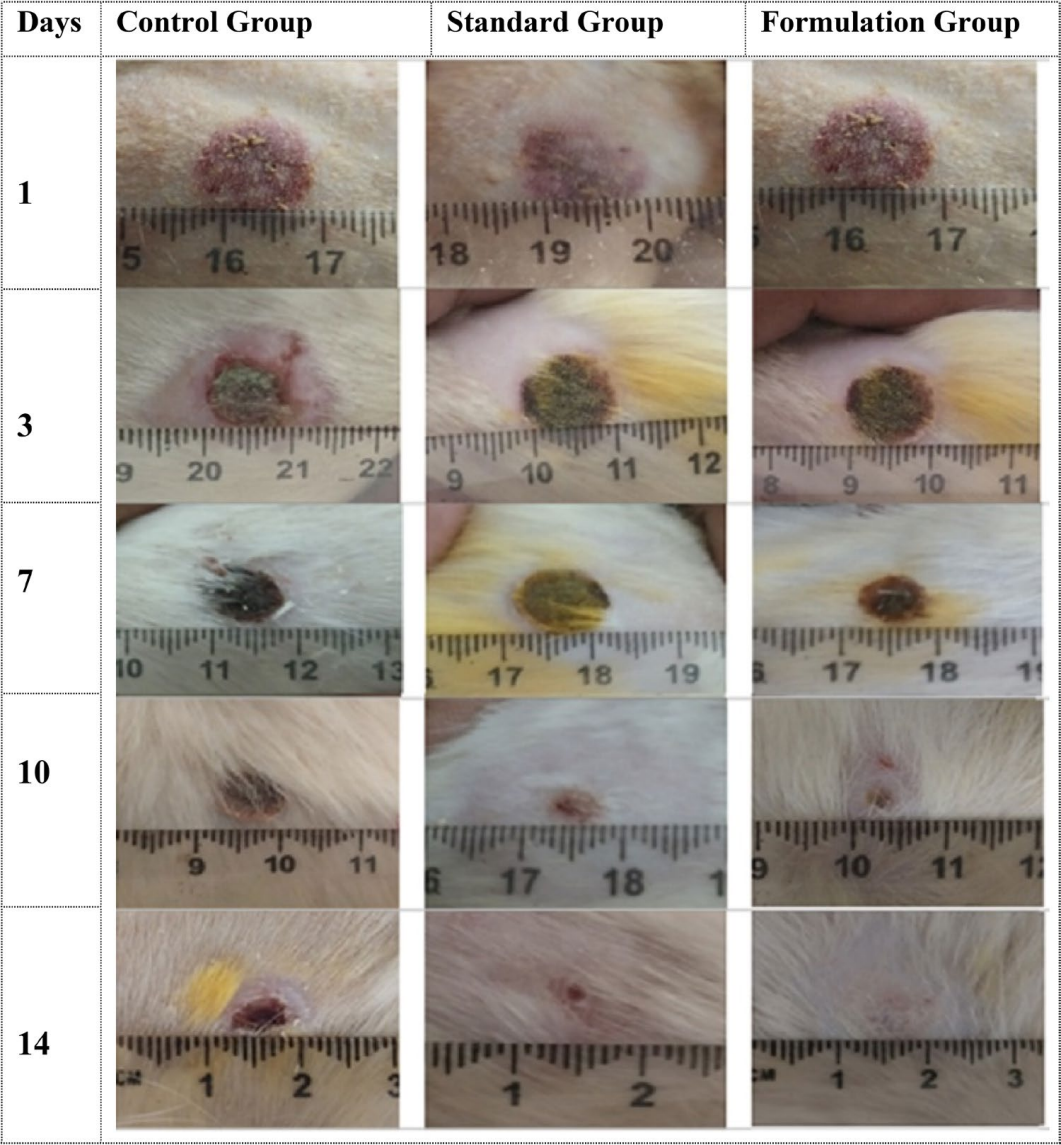
Photographs of burn wounds in rats taken on 1^st^, 3^rd^, 7^th^, 10^th^, and 14^th^ day of dosage form application in different rats groups (i.e., CG, SG and FG).

Burn wound contraction was expressed and measured in percentage of the total wound regenerated area versus the original burn wound area. Which gives information about wound shape distortion and wound total area shrinkage in various animal groups. All three rat groups have 0% of wound contraction on day zero. Over the period of time, the FG showed significant contraction of wound as compared to CG and SG (*p* < 0.05) (Table 3). On day 3, FG showed prominent wound contraction (42.06 ± 1.39%) as compared to SG (24.76 ± 3.8%) and CG (17.2 ± 1.35%). Similarly, wound contraction difference was maintained at day 7 for FG, SG, and CG which were 74.23 ± 1.9%, 68.76 ± 4.72%, and 40.92 ± 5.32% respectively, day 10 (FG: 84.3 ± 3.51%, SG: 78.5 ± 4.9%, and CG: 65.2 ± 6.73%), and day 14 (FG: 98.13 ± 1.57%, SG: 91.78 ± 6.52%, and CG: 76.93 ± 7.21%). At day 14, the difference of groups was narrow because of wound recovery and all wounds were trending wound closure, especially FG and SG. The wound pertaining to FG of rates showed complete wound healing, while a small open wound area was observed in CG and SG. There was a significant difference between treated group and control group wounds throughout the wound recovery period (Figure 4).

**Table 3.** Percentage wound contraction of burn wounds on 3^rd^, 7^th^, 10^th^, and 14^th^ day (mean ± SD; *n* = 6).

Days	Control group (%)	Standard group (%)	Formulation group (%)
3	17.2 ± 1.35	24.76 ± 3.8	42.06 ± 1.39
7	40.92 ± 5.32	68.76 ± 4.72	74.23 ± 1.9
10	65.2 ± 6.73	78.5 ± 4.9	84.3 ± 3.51
14	76.93 ± 7.21	91.78 ± 6.52	98.13 ± 1.57

#### 3.2.3. FTIR burn wound analysis

Environmental elements such as water and carbon dioxide can impact infrared spectra. The water vapor spectrum in the amide bands areas (1600–1800 cm^−1^) in particular influence FTIR spectra. We reduce the interval between background measurements and purge the FTIR equipment with dry air associated with desiccants to reduce the influence of background peaks associated with water vapor and carbon dioxide. Furthermore, according to Diem et al, a principal component analysis on the full data set can be used to validate residual changes associated with rot vibrational transitions of the water molecule (Diem, 2018).

Animals were divided into 3 groups; i.e., CG, SG, and FG. Each group consisted of five animals, and samples were collected from each group on day 3, day 7, and day 14 for molecular investigation using FTIR spectroscopy to assess the burn wound recovery. FTIR spectra provide information of lipids, proteins, and nucleic acid (Castro et al., 2021; Gul et al., 2022).

In CG, the absorbance intensity of amide I band was increased at 1632 cm^−1^ and 1635 cm^−1^, and also shifting from single peak to double peak on day 7^th^ and again into single (1635 cm^−1^) but broader peak on day 14^th^. Here, 1632 cm^−1^ peaks represent C = C uracil and C–O stretching, and 1635 cm^−1^ peak was due to the β sheet structure of amide I. The absorbance of amide I increased from 0.13 to 0.27 in 14 days of recovery. The peak at 1553 cm^−1^ was due to absorbance intensity of α sheet of amide II and its absorbance intensity also increased from 0.09 to 0.18 in 14 days. An asymmetric CH_3_ bending mode of the methyl group of proteins (1455 cm^−1^), intensity of these peaks increases as the treatment progresses. Amide III band at around 1239 cm^−1^ increase with the progression of wound recovery was due to PO^−2^ stretching.

In SG, the information in IR spectra is the combination of different bio-molecules like carbohydrates, lipids, proteins, and nucleic acids. As the treatment progressed, the two peaks; 1632 cm^−1^ and 1635 cm^−1^ emerged as a single peak. The merging of two peaks into a single peak (1635 cm^−1^) was due to amide 1. Stretching C = C, C = O, C5 methylated cytosine, NH_2_ (1635 cm^−1^), uracil, and nucleic acid peaks due to breathing mode and carbonyl stretching. The amide II band at 1553 cm^−1^ showed peak shifts as the healing process progressed. It might be due to stretching C = N, C = C, and methyl deformation, respectively. At 1028 cm^−1^, an increase in a band was observed as the healing progress to full recovery. The 1028 cm^−1^ shift was due to oligosaccharide C–OH stretching, 2-methyl mannoside, mannose and mannose 6 phosphate. Peak at 1239 cm^−1^ represents asymmetric PO^−2^ stretching (amide III). An increase in absorbance and peak area in amide 1, amide II and amide III as the healing progresses demonstrate that change in stratum corneum occurs due to the weakening of hydrogen bond density and increase in collagen concentration.

In FG, a similar pattern of absorbance peaks was observed. The absorbance intensity of amide I band at 1632 cm^−1^ and 1635 cm^−1^ increased as treatment progressed and merged into a single but broader peak. The 1635 cm^−1^ absorbance peak represents the β-sheet structure of amide I and the 1553 cm^−1^ absorption peak was due to the α-sheet of amide II. ATR-FTIR data suggests that increase in amide I, amide II and amide III were observed with treatment progression.

**Figure 5.**
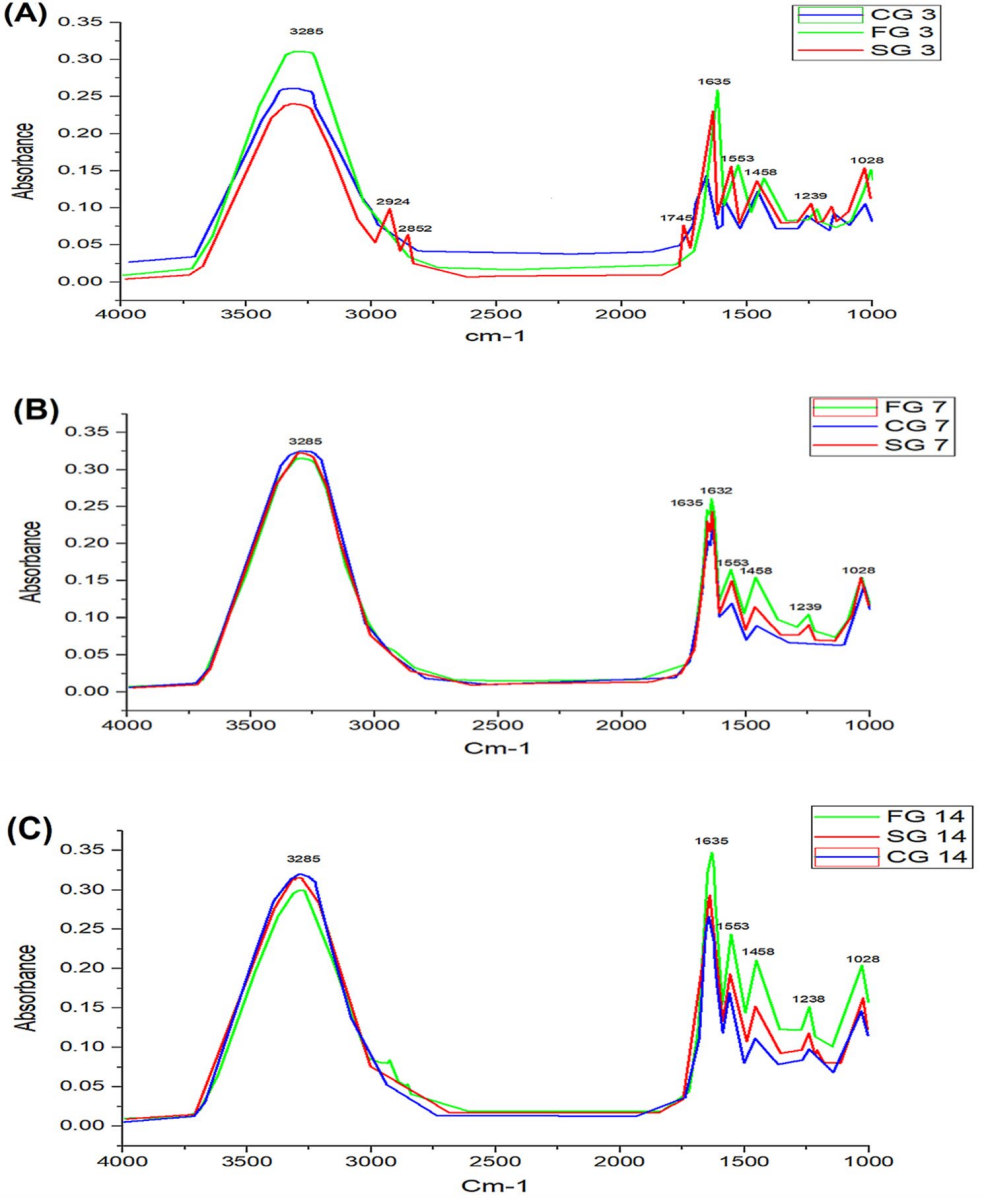
FTIR comparative graph on day 3 (A), day 7 (B), and Day 14 (C). Wound samples taken from formulation groups (FG), control group (CG) and standard group (SG).

**Figure 6.**
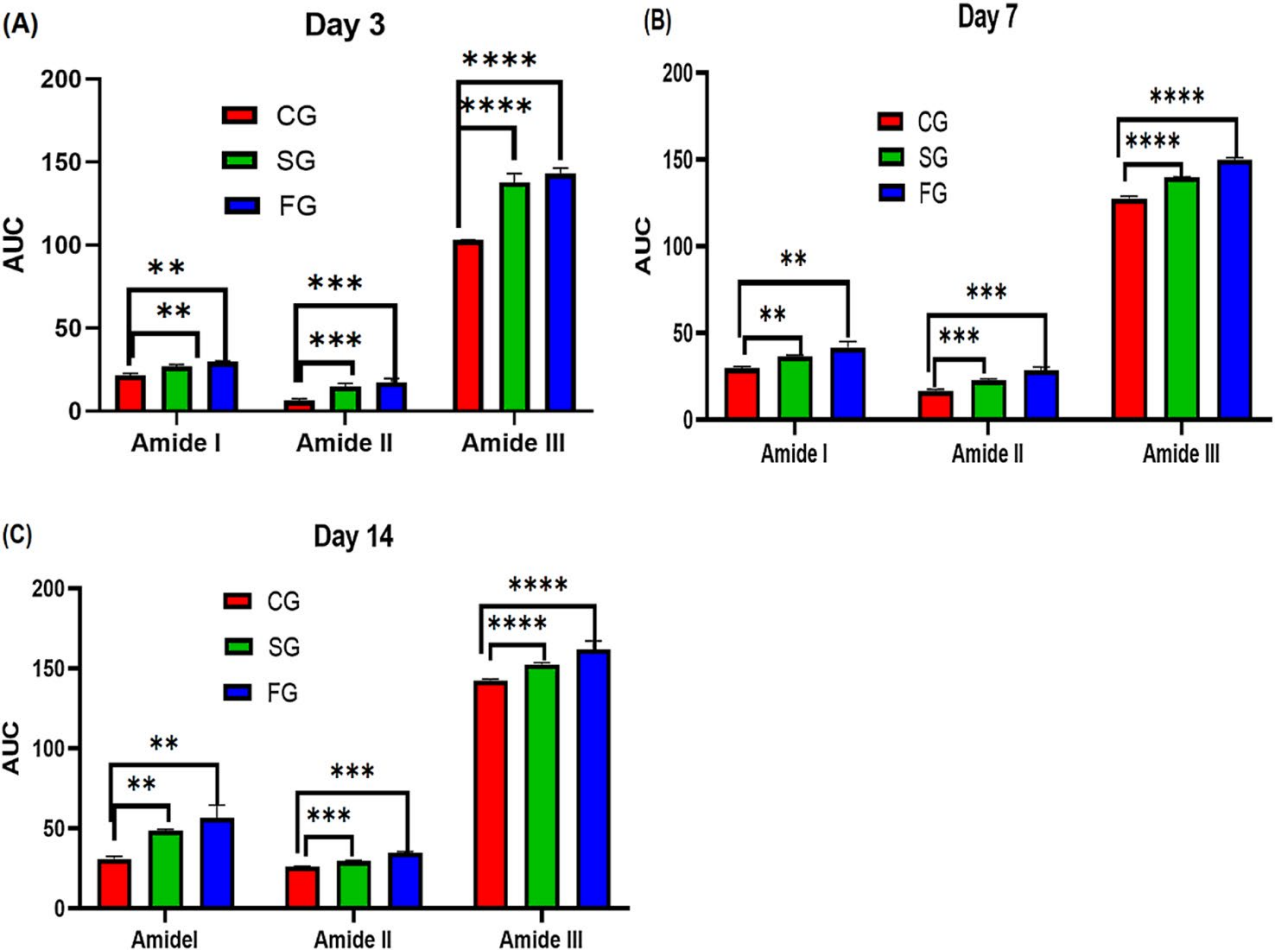
(A) to (C) shows the CG, SG and FG amide I, Amide II and Amide III concentration on day 3, day 7 and day 14. Column represents mean values; a bar represents significance level (** <0.01, *** < 0.001, **** ≤0.0001).

**Figure 7.**
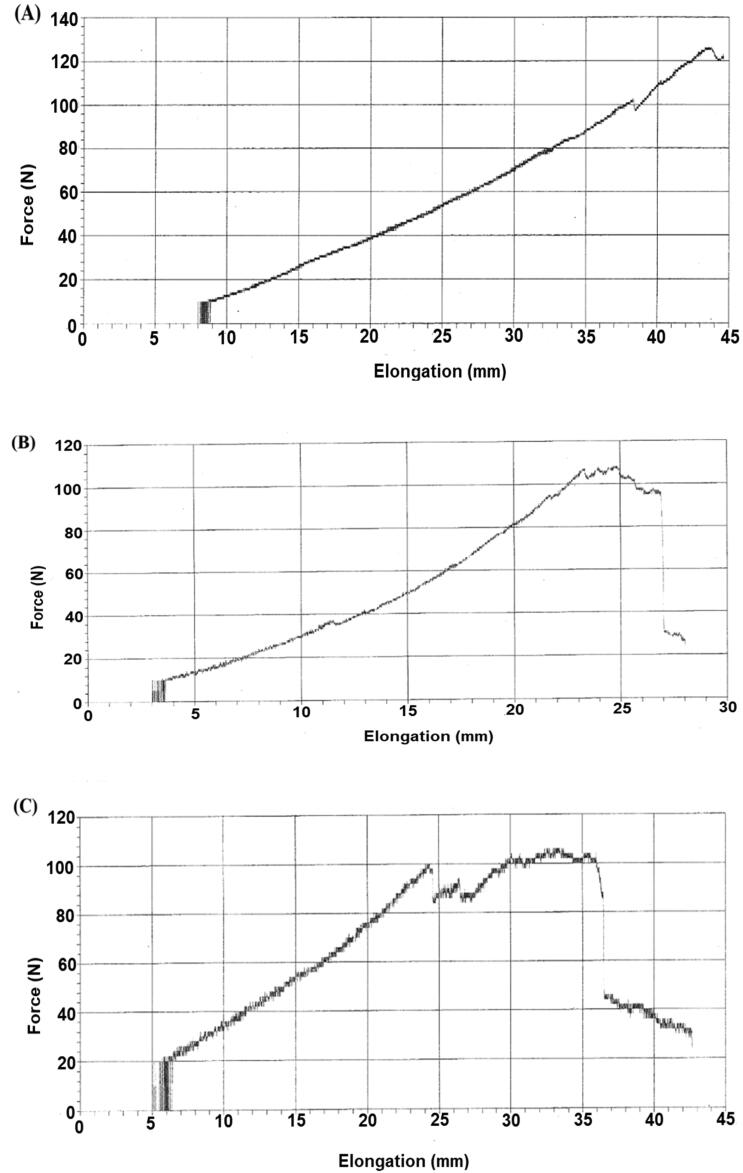
The peak load of FG (A), SG (B), and CG (C) on day 14^th^ of burn wound healing.

Results obtained using FTIR spectroscopy revealed biochemical information of all the 3 groups; CG, SG, and FG (Figure 5). High content of lipids, proteins, and carbohydrates were observed in post burn injuries which indicates vehement metabolic activities in burn injury and are linked with the cascade of processes triggered by burn wound, and also activation of biological events allied with hemostasis phase of wound site (Castro et al., 2021). In our study, we demonstrated the use of FTIR in each phase of the healing process for biochemical information assessment. Although, FTIR is not capable of providing information similar to that of molecular test but FTIR findings can establish FTIR imaging, which is capable of providing data regarding tissue repair of burn wounds and also gives information regarding biological changes triggered by second-degree burn wounds. ATR-FTIR analysis was performed on days 3, 7, and 14, and the results were clear from the peaks. On day 3, the absorbance peaks for amide I, amide II and amide III were small as compared to day 7 and day 14 as shown in Figures 6 and 7. The absorbance peaks suggest that the greater the absorbance of a specific peak, the greater will be the concentration of said component (Baker et al., 2014).

Figure 5(A–C) demonstrates the complete spectrum data set using this chemometric quality technique. This figure shows that there is no substantial negative impact of water vapor on spectral quality over the 1600–1800 cm^−1^ range when comparing CG, SG, and FG on day 3, day 7 and day 14 FTIR spectra. Figure 5(A–C) shows the average spectra of post-burn injuries throughout a 4000–400 cm^−1^ range. The peak positions between the spectra do not reveal changes in the arrival and disappearance of infrared bands, as shown in Figure 5(A–C).

Using the AUC, an assessment of the potential consequences of heat damage on overall biochemical status was made. Figure 6 shows the AUC bands of CG, SG and FG burn wounds. The AUC gradually augments for amide I, amide II, and amide III as the time progresses after thermal injury. In Figure 6, the significant difference between the CG, SG and FG can be seen. There were substantial differences between the AUC values obtained at 1635 cm^−1^, 1553 cm^−1^, and 1458 cm^−1^ when compared. These bands could be linked to an increase in the relative amount of protein tissue, implying a link between collagen activities and wound healing. The molecular and physiological foundations of wound healing may lead to fibroblast proliferation and protein accumulation in the extracellular matrix. The spectral markers of tissue repair mechanisms could be the high difference of these molecular bands during the healing process (Storey & Helmy, 2019). When the skin’s integrity is challenged by thermal injury, the healing reaction kicks in. As a result, infection could potentially stymie the healing process. In comparison to current diagnostic techniques, recent investigations have proven the importance of low-cost vibrational spectroscopic approaches for analyzing spectrum markers that are label-free.

### 3.3. Tensile strength

Tensile strength, also known as peak load, was calculated and compared for all three groups. Tensile strengths of recovered skins were performed on day 14 of the CG, SG, and FG using UTM (Figure 7). The peak load, break load, and breaking elongation of FG were higher than the SG and CG. The peak load was 126 ± 0.23 N for FG, 108 ± 1.7 N for SG, and 53 ± 4 N for CG. Break load of CG, SG, and FG was 24 ± 3 N, 71 ± 1.2 N, and 119 ± 4 N, respectively, while, breaking elongation for CG, SG, and FG were 28 ± 3 mm, 35 ± 2 mm, and 44.6 ± 2 mm, respectively.

## 4. Conclusion

The nanoparticulate system is an exciting platform with many applications in the wound healing process. In the last decade, many researchers have investigated the development of novel therapeutic modalities which are capable of enhancing wound management with no or limited scar formation. In this work, ACR-PCL-NE was prepared for burn wound healing using double emulsion solvent evaporation technique. The results demonstrated that the emulsion was stable with desired physicochemical properties such as particle size, polydispersity index, surface morphology, pH, etc. The pH of formulation was compatible with the skin so it can be applied with ease without skin irritation. The particle size, zeta potential, and polydispersity index of ACR-PCL-NE were in acceptable range when observed after 90 days with no phase separation. Finally, the rat was used as an animal model to evaluate the efficacy of prepared formulation using different assessment techniques. The *in vivo* results of percentage wound contraction, peak load, break load, breaking elongation, and ATR-FTIR studies of burn wound suggest the effectiveness of ACR-PCL-NE over commercially available marketed drug. The *in vivo* study results revealed that the FG has high wound healing potential as compared to the SG and CG. From our findings it can be concluded that this formulation can be successfully used for wound healing process enhancement and efficacy of this drug can be evaluated in human volunteers in future. Also, ATR-FTIR studies in the future can be used to evaluate wound healing phases beside of histological analysis, which is a more precise way in evaluation the healing process and small sample size of the skin as compared to histological analysis.
